# Modeling Effects of Local Extinctions on Culture Change and Diversity in the Paleolithic

**DOI:** 10.1371/journal.pone.0015582

**Published:** 2010-12-17

**Authors:** L. S. Premo, Steven L. Kuhn

**Affiliations:** 1 Department of Human Evolution, Max Planck Institute for Evolutionary Anthropology, Leipzig, Germany; 2 School of Anthropology, University of Arizona, Tucson, Arizona, United States of America; University College London, United Kingdom

## Abstract

The persistence of early stone tool technologies has puzzled archaeologists for decades. Cognitively based explanations, which presume either lack of ability to innovate or extreme conformism, do not account for the totality of the empirical patterns. Following recent research, this study explores the effects of demographic factors on rates of culture change and diversification. We investigate whether the appearance of stability in early Paleolithic technologies could result from frequent extinctions of local subpopulations within a persistent metapopulation. A spatially explicit agent-based model was constructed to test the influence of local extinction rate on three general cultural patterns that archaeologists might observe in the material record: total diversity, differentiation among spatially defined groups, and the rate of cumulative change. The model shows that diversity, differentiation, and the rate of cumulative cultural change would be strongly affected by local extinction rates, in some cases mimicking the results of conformist cultural transmission. The results have implications for understanding spatial and temporal patterning in ancient material culture.

## Introduction

The gradual pace of change and relatively low level of diversification in early stone tool technologies is deeply puzzling to archaeologists and paleoanthropologists. It is widely agreed that both the forms of artifacts and the methods used to make them changed slowly and varied little during the Lower and Middle Paleolithic when compared to later periods [1]–[4]. Stone tools, even comparatively complex ones such as handaxes and Levallois flakes, were produced in a limited array of forms for hundreds of thousands of years. On the other hand the Lower and Middle Paleolithic were not completely static. There is a noteworthy diversity in methods for producing flakes and blades, particularly in the Middle Paleolithic (e.g., [5]–[7]). Site stratigraphic or regional sequences may contain a succession of assemblages with different technological characteristics, demonstrating a sort of change through time [6], [8]. A better description of the situation is that truly new technological behaviors appear very infrequently. Instead, variability across space and through time appears attenuated. The same or similar artifact forms and methods of production recur again and again at different times and places. Moreover, it has been difficult to link documented variation in Middle Paleolithic artifact diversity or complexity to environmental factors [9], [10] or even hominin species.

A range of cognitively based explanations has been offered for the relative lack of novelty and change in Lower and Middle Paleolithic technologies. Innovation is considered to be a key source of change in human behavior and culture [11], [12] and some researchers argue that the apparent stability of Lower and Middle Paleolithic material culture is rooted ultimately in the inability to innovate on the part of the hominins that produced the artifacts [13]–[15]. An opposing position holds that these individuals actually resisted change. From this perspective, highly persistent cultural traditions reflect what is in essence an extreme form of biased cultural transmission (e.g., [16], [17], but see [18]).

Neither of these two classes of explanation is wholly satisfactory. Besides relying on what are—for now at least—otherwise undetectable cognitive traits to explain patterns of technological evolution, they are not entirely consistent with the archaeological evidence. Lower and Middle Paleolithic hominins were capable of at least some innovation, of solving novel adaptive problems by altering their behavior. They learned to cope successfully with a wide range of environments, particularly after 800,000 years ago when they began to establish populations in the northern temperate zones (see recent assessments in [19], [20]). They also were able to exploit stones with very different working properties to produce tools, and to maintain a supply of tools and raw materials even when suitable stone was scarce. The proposition that early humans could maintain highly conservative traditions over such long spans of time and over such a large area implies a very high level of fidelity in cultural transmission. Although this possibility cannot be dismissed entirely, it does presuppose a social or cognitive mechanism capable of maintaining strong conformism over thousands of generations in pre-literate societies.

More recently, researchers have turned to demographic factors, effective population size in particular, to explain the appearance, diffusion, and survival of novel cultural ideas over the course of human evolution. An important conclusion is that beneficial new ideas are more likely to survive and spread in larger, more thoroughly interconnected populations. Shennan and colleagues [21], [22] argue that the “creative explosion” of the Eurasian Upper Paleolithic and African late Middle Stone Age is at least partly a function of larger and more robust populations. This implies that the apparent resistance to innovation and directional change in earlier populations may in turn be a reflection of smaller effective population sizes, which would intensify the effects of drift-like processes in eliminating novel and rare cultural variants, even if they had adaptive value [23]–[25]. Interconnectedness between individuals and groups, mediated by social and cognitive factors as well as demography, would also play a major role in the spread of beneficial innovations [26].

In this paper we use a spatially explicit agent-based model to explore the effects of another demographic factor, localized extinction, on patterns of cultural change and diversity. We investigate whether the appearance of temporal stability and geographic homogeneity in early Paleolithic cultures could result from frequent extinctions of local subpopulations within a persistent metapopulation. The model explicitly considers selectively neutral traits rather than ones that differentially affect fitness. Consequently we treat innovations and copying errors as equivalent. There are two reasons for this. The first is empirical. Although artifacts certainly had an adaptive value, much of what we know about diversity in early Paleolithic behavior concerns phenomena such as subtle variations in how stone was worked into usable tools. There is little evidence that these culturally transmitted variants would have contributed significantly to the differential fitness of their bearers. Some variants may have had beneficial effects under certain conditions, but this cannot be assumed [27]. As noted above, it is also difficult to identify environmental correlations with the composition and forms of Middle Paleolithic toolkits [9], [10]. The second reason is heuristic. Modeling fitness effects (whether environmental or social) could easily be made either to force change or to restrict it. An assumption of neutrality is less restrictive.

There are also good reasons to believe that populations in Pleistocene Eurasia experienced frequent local extinctions or stochastic loss of small subpopulations. Genetic evidence suggests that Neanderthals at least maintained comparatively low effective population sizes and were fragmented into small subpopulations [28]–[30]. It is now well established that Neanderthals in particular fed high in the trophic pyramid, and were as carnivorous if not more carnivorous than later *Homo sapiens* [31]–[33], and this could have contributed to subpopulation instability [34]. Carnivore species are known to experience higher rates of extinction than herbivore species [35]–[38]. In part this is because carnivores maintain lower population densities than herbivores, and small populations are more vulnerable to chance events. It is also apparent that human populations in temperate Eurasia, including recent ones [39], expanded and contracted in response to major climate cycles. The retreat of Middle Paleolithic populations from Northern Eurasia during glacial periods most likely resulted from localized extinctions in the least hospitable areas rather than from long-range population movement [20], [40].

We conducted a series of experiments with the agent-based model to test whether rates of local group extinction could inhibit culture change and regional differentiation. We tested the influence of local extinction rate on three general patterns that archaeologists might observe in the material record: total cultural diversity, cultural differentiation among spatially defined groups, and the rate of cumulative cultural change. The results of these experiments show that local extinctions can have interesting effects on culture change and diversity.

## Methods

### The model

We employ a spatially explicit agent-based model to simulate structured populations of constant size and density. The model includes just two classes of agents: individuals and groups. These classes are hierarchical in the sense that the properties of a group are defined by the properties of the individuals it contains. Individuals serve as little more than vehicles for the cultural transmission of selectively neutral variants. Cultural variants are represented by integers. Groups serve to structure the metapopulation and to help operationalize local extinctions. Each group (*n* = 100) occupies a single cell on a 2-dimensional 10×10 grid-based lattice, which is wrapped around a torus to avoid edge effects. Each group can contain no more than *N* = 25 individuals.

The model has two important experimental parameters. The first, *e*, provides the probability that each group suffers local extinction during any given time step. In other words, *e* provides the proportion of groups on average that succumb to local extinction during each time step. The second parameter, *μ*, provides the probability that a naïve individual makes a copying error during cultural transmission. Our methodology allows us to systematically investigate whether the frequency of local extinctions (*e*) affects total cultural diversity, the degree of group differentiation, and the rate of cumulative cultural change in idealized metapopulations while controlling for copying error rate (*μ*).

Each model time step represents a single, non-overlapping generation (or alternatively a single, metapopulation-wide round of cultural transmission) and involves four stages.

#### Stage 1 (local group extinction and recolonization)

Life for the group can be precarious. The fate of the entire group can rest on factors as unpredictable as the duration of a cold snap, a seasonal shortfall of prey, or the health of its hunters. Thus, local extinction is modeled as a stochastic process that each group faces at the start of every time step. When a group experiences local extinction, all of its members are removed from the simulation immediately. To fill the void left by a local extinction event, half of the members (rounded to the nearest whole number) from a randomly chosen group from the Moore neighborhood “colonize” the empty cell. In other words, each local extinction event entails the disappearance of one group *and* the fissioning of an adjacent group. In our view this pattern of recolonization from groups in adjacent areas is a closer approximation of dynamics of forager groups in space than is randomized repopulation from any group in the grid regardless of distance.

In the vast majority of cases, the group chosen to provide the “colonizers” for the empty cell possesses the full complement of 25 individuals. In such cases 13 individuals move to the empty cell and 12 remain in their current location as a result of fissioning. However, the group randomly chosen to fission may have already been involved in an extinction and recolonization event. These groups consist either of the “colonizers” or the members that remained behind. The same fission rule applies to groups with fewer than 25 individuals: half of the individuals (rounded to the nearest whole number) in the group move to the empty cell while the rest remain in their current group. Thus, while the size of each colonizing party can range from 1 to 13 individuals, the modal size is 13 individuals.

#### Stage 2 (create “offspring” generation)

A new generation of individuals is created to take the place of the previous generation. Each group receives *N* = 25 naïve individuals.

#### Stage 3 (cultural transmission)

During this stage, cultural variants are transmitted from what remains of the experienced “parental” generation to the newly created and naïve “offspring” generation. In our model, cultural transmission occurs *within* groups only. The model implements two different mechanisms of social learning: unbiased and conformist biased cultural transmission. With unbiased cultural transmission, each naïve offspring learns (i.e., copies) the variant expressed by an individual that it chooses randomly (with replacement) from among the parental generation in its group. With conformist biased transmission, each offspring copies the cultural variant that is most commonly displayed (i.e., the modal variant) among members of the parental generation in its group. In cases where the parental generation of a group displays more than one modal variant, each naïve individual randomly selects one of the modal variants to copy.

Cultural transmission is noisy. The probability that each naïve individual makes a mistake when copying a variant is given by *μ*. Copying errors made during cultural transmission provide the only source of new variation in this model. The results presented here allow for bidirectional errors in replication: that is, a mistake in copying results in a variant that is one step higher or lower than the parental variant. As a result, cultural variants embark on a symmetric one-dimensional random walk with a step length of 1. We feel that this is a reasonable first approximation of phenomena such as lithic technology for two reasons. First, new technological innovations or tool forms commonly build incrementally on earlier ones. Second, older technologies and tool forms can and do reappear. It is worth noting that we also ran a version of the model that included a unidirectional (i.e., forward only) representation of copying errors, and outcomes were similar except for *F_ST_*, a measurement for which the assumption of unidirectional copying errors can be problematic.

#### Stage 4 (remove “parental” generation)

All of the remaining members of the parental generation are removed from the simulation following cultural transmission. At the end of this final stage, the metapopulation (and each of the groups) is the same size and density as at the start of the time step. The assumption that groups grow from a relatively small number of colonizers (or those that remain behind) to *N* within a single generation is commonly made in metapopulation models. This rapid repopulation is important in that we want to examine the effects of localized extinctions independently of changes in census population size or density. In any real situation high levels of local extinction would obviously reduce the census population size and cause regional densities to vary. The consequences of population size and local extinctions for cultural diversity and change would likely be additive. Therefore, by assuming a constant census population size, we bias our model against the hypothesis that local extinctions decrease cultural diversity, group differentiation, and the rate of change.

The source code for our NetLogo [41] model is provided as a supplementary file (Text S1). A complete model description following the standard protocol for agent-based models [42] is also provided as supplementary material (Text S2).

### Equilibrium vs. non-equilibrium conditions

**Figure 1:**
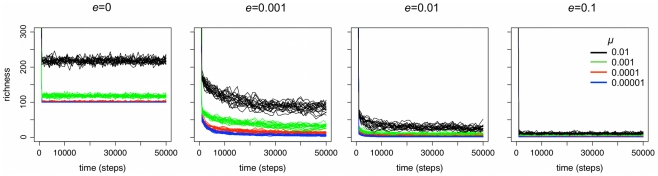
Equilibrium conditions for all combinations of *μ* and *e* tested here. Richness decreases quickly from an initial value of 2500 and cultural diversity reaches equilibrium by the 50,000^th^ time step in all populations. Each line represents the results of a unique simulation run.

It is preferable to assess demographic effects on diversity in systems that are at equilibrium. In the absence of selection, cultural diversity reaches equilibrium when the rate that new variants are introduced via copying errors matches the rate at which unique variants are lost to drift. The number of generations required to reach equilibrium varies with population size, copying error rate, population structure, and, in our model, the frequency of local extinctions. When the copying error rate is very low and there are no local extinctions and the starting population is perfectly homogeneous, many hundreds of thousands of generations must pass before cultural diversity approaches equilibrium. Metapopulations reach equilibrium more quickly if every individual displays a unique cultural variant (in this case, an integer chosen randomly from between 0 and 10^8^) at the start of the simulation. When starting with heterogeneous metapopulations, 50,000 time steps are sufficient for cultural diversity to reach equilibrium for all combinations of *μ* and *e* tested here (Figure 1).

In addition to data collected under equilibrium conditions, we analyze data collected from metapopulations that are not at equilibrium. There are two reasons for this. Starting from heterogeneous metapopulations complicates the task of measuring the effects of local extinctions on distance-based measures of group differentiation and cultural change. A less pragmatic but no less important issue is that, while equilibrium serves as a useful convention for providing a clearer understanding of the effect of local extinctions on some diversity measures, it is not meant to reflect reality. It is not known whether cultural diversity ever reached equilibrium in Lower and Middle Paleolithic societies, nor is this assumption necessary here. The non-equilibrium data were collected after 100,000 time steps from metapopulations in which every individual displayed a cultural variant of “0” at the start of the simulation. This complementary set of experiments allows us to study the effects of local extinctions on diversity measures, distance-based measures of group differentiation, and rates of cultural change as a function of time.

### Measuring total cultural diversity

Archaeologists use a variety of indices to quantify and compare the diversity of “types”—categorical variants of form, technological mode, raw material, etc.—observed in archaeological datasets. They range from relatively simple measures, like richness (the number of unique variants) and evenness (the variability in the relative frequencies of unique variants), to more sophisticated measures that account for both, such as Simpson's *D* and Shannon's *H*′. Shannon's *H*′ is calculated as follows:
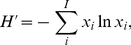
(1)where *I* is the number of unique variants observed (i.e., richness) and *x_i_* is the relative frequency of the *i*-th variant. *H*′ is bound by 0 (lower) and ln *I* (upper). In the context of our model, *H*′ = 0 indicates that every individual in the metapopulation displays the same cultural variant. *H*′ increases as a function of both the richness and evenness of cultural variants.

Richness and Shannon's *H*′ do not take population structure into account. Population geneticists use other measures, such as *H_T_*, to analyze genetic diversity in structured populations. Because we have modeled the cultural trait after a microsatellite locus, we can use *H_T_* to quantify cultural diversity in our artificial populations. Imagine a metapopulation composed of *K* groups. Again, let *I* be the total number of unique cultural variants displayed in the metapopulation. Let *x_ki_* represent the relative frequency of the *i*-th cultural variant as observed in the *k*-th group (note that *x_ki_* = 0 when *i* is not displayed by at least one of *k*'s members). Following Nei and Kumar [43], *H_T_* is defined as:
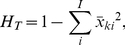
(2)where 
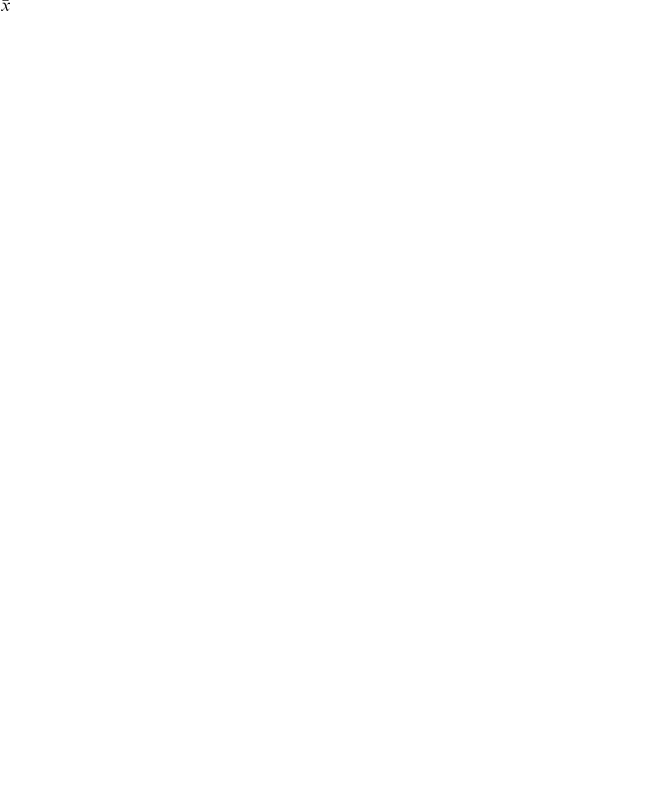
 *_ki_* is the average of *x_ki_* over all groups. *H_T_* is bound by 0 and 1. In the context of our model, *H_T_* = 0 indicates that every individual in the metapopulation displays the same cultural variant.

### Measuring differentiation among groups

To investigate whether frequent local extinction—independent of rates of copying errors—could affect levels of regional differentiation in Paleolithic societies, we apply two measures of group differentiation to our simulated data: (1) the mean cultural distance between the modal variant of each group and the modal variants of all other groups (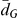) and (2) the proportion of total cultural diversity explained by differences between groups (*F_ST_*).

The mean cultural distance between the modal variant of each group and the modal variants of all other groups can be calculated as follows:
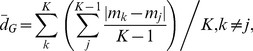
(3)where *m_j_* and *m_k_* represent the modal cultural variants of the *j*-th and *k*-th groups, respectively. Low variability among the modal variants displayed by different groups results in 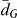 near zero. As variability among modal variants increases, so does 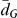. We present and discuss the 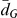 results collected from non-equilibrium conditions only, because this measure is not meaningful for a highly polymorphic starting condition when *e* is low.

Population geneticists have developed more sophisticated methods for quantifying group differentiation in structured populations. Their methods deal not with distances between groups' modal allele frequencies, but with how metapopulation-level variation in allele frequencies is partitioned within and between subpopulations. Wright [44], [45], [46] developed three parameters—collectively referred to as F-statistics—for measuring the deviations from Hardy-Weinberg equilibrium in genotype frequencies in structured populations. One of these parameters, *F_ST_*, provides a useful measure of differentiation among local subpopulations. Nei [47] generalized *F_ST_* such that it can be applied to diploid or nondiploid loci that have multiple (i.e., more than 2) alleles and can be passed by sexual or asexual reproduction. Thus, Nei's *F_ST_* (also known as *G_ST_*) is appropriate for measuring group differentiation in our simulated populations.

Obtaining Nei's *F_ST_* involves just three steps: calculate the average diversity of the entire population, calculate the average diversity found within groups, and then subtract the proportion of total diversity explained by within-groups diversity from 1. The result—the proportion of total diversity explained by differences between groups—provides a measure of group differentiation in a structured population. We have already discussed the method for measuring the average cultural diversity of a structured population (*H_T_*, see Equ. 2). Next is the task of measuring the within-groups component of average cultural diversity (*H_S_*). Following Nei and Kumar [43]:
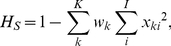
(4)where *w_k_* is the size of the *k*-th group relative to the metapopulation and *K*, *I*, and *x_ki_* are as defined above. Because all groups are of equal size (*N* = 25) at the time of data collection, *w_k_* = 1/*K*. Calculating *F_ST_* from *H_T_* and *H_S_* is straightforward:
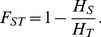
(5)


Note that *F_ST_* makes little sense when *H_T_* = 0. We found this to be the case for many of the simulated populations characterized by a relatively low copying error rate (*μ*≤0.0001) and a relatively high frequency of local extinctions (*e*≥0.01). For this reason, we present the *F_ST_* results of the two higher copying error rates only.

### Measuring rates of cumulative cultural change

To investigate the consequences of local extinctions on the rate of cumulative cultural change for a given *μ*, we compare the number of copying errors that the metapopulation accumulates under different values of *e*. Recall that every non-equilibrium simulation is initialized with a homogeneous metapopulation in which all individuals display the value “0” as their cultural variant. Also recall that copying errors can only increase or decrease the value of a cultural variant by 1 (i.e., a copying error cannot result in a value of “5” unless the target value was either “4” or “6”), and that we hold the number of cultural transmission events constant. Thus, a larger accumulation of copying errors in a metapopulation signifies a faster rate of cumulative culture change per transmission event.

Let us consider the dynamics of neutral culture change in a structured population with unbiased cultural transmission and a bidirectional model of innovation. In the absence of local extinctions (*e* = 0), the metapopulation accumulates copying errors at a rate proportional to *μ*. The actual rate of cumulative culture change is less than *μ* because of “back innovations” and the fact that many variants are lost to the sampling effects associated with randomly choosing teachers within groups. Stochastic local extinctions may also serve to remove cultural variants from the metapopulation. While it is apparent that local extinctions may further inhibit the accumulation of copying errors in a structured population, understanding the magnitude of this effect requires systematic investigation.

The dynamics of cumulative culture change in a population with conformist transmission are quite different. In this case, the variants introduced via copying errors are lost immediately so long as copying errors are not commonplace (*μ*<0.5). This is not because of drift, but because the frequency-dependent mechanism of conformist cultural transmission actively selects *against* all non-modal variants. Local extinctions are unlikely to affect the rate of cumulative change in the presence of conformist transmission because drift is weak relative to the bias introduced by copying the most common variant in the group.

The number of copying errors that accumulate in any group during the course of a non-equilibrium simulation can be assessed by the absolute value of the group's modal cultural variant. Because all individuals display a cultural variant of “0” at the start of each non-equilibrium simulation, we refer to the value of a group's modal cultural variant as its *distance from ancestral* (*d_A_*). We use the absolute value of the group's modal variant rather than the variant with the maximum absolute value in order to conform to the normative way archaeologists most often perceive and describe the material record. Perceptions of variation within and among Paleolithic assemblages are strongly biased in favor of the most common or most “important” technological variants. Rare artifact classes or unique technological procedures may be systematically reported but are seldom accounted for in large-scale syntheses and regional comparisons. The maximum rate of cumulative change per cultural transmission event in a structured metapopulation is represented by the maximum |*d_A_*| value found among its subpopulations (*d_A max_*). Note that *d_A max_* does not provide a suitable proxy for the rate of cumulative cultural change if metapopulations are initialized with maximum heterogeneity.

## Results

The results of the model show clearly that rates of local extinction could influence total diversity, group differentiation, and rates of long-term cumulative change. These findings are summarized below.

### Local extinction decreases total cultural diversity

**Figure 2:**
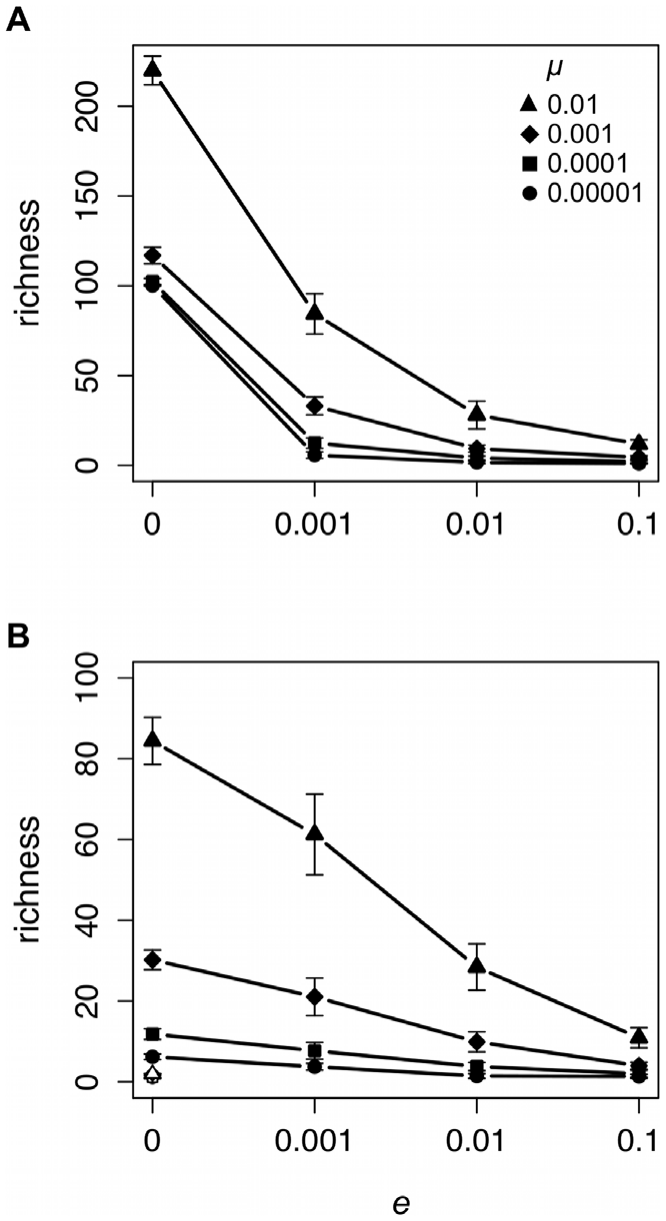
Local extinction rate (*e*) affects richness under equilibrium (A) and non-equilibrium (B) conditions. Each data point provides the mean ±1 standard deviation of 20 unique simulated populations. Black symbols represent data collected from populations with unbiased cultural transmission and white symbols data collected from populations with conformist cultural transmission.

One of the simplest measures of total diversity is richness (Figure 2). As one would expect, richness increases with *μ*, although the magnitude of this effect decreases as *e* increases. More importantly, *e* has a significant effect on richness for all values of *μ* tested under equilibrium (Kruskal-Wallis H-test results: *μ* = 0.00001: *χ* ^2^ = 72.54, P<0.001; *μ* = 0.0001: *χ* ^2^ = 73.26, P<0.001; *μ* = 0.001: *χ* ^2^ = 74.06, P<0.001; *μ* = 0.01: *χ* ^2^ = 74.12, P<0.001) and non-equilibrium (*μ* = 0.00001: *χ* ^2^ = 67.87, P<0.001; *μ* = 0.0001: *χ* ^2^ = 69.82, P<0.001; *μ* = 0.001: *χ* ^2^ = 72.29, P<0.001; *μ* = 0.01: *χ* ^2^ = 73.91, P<0.001) conditions. Richness decreases as *e* increases. Our results also suggest that the combination of unbiased transmission and frequent local extinctions can maintain a similar number of unique cultural variants as conformist biased cultural transmission in the absence of local extinctions (Figure 2b).

**Figure 3:**
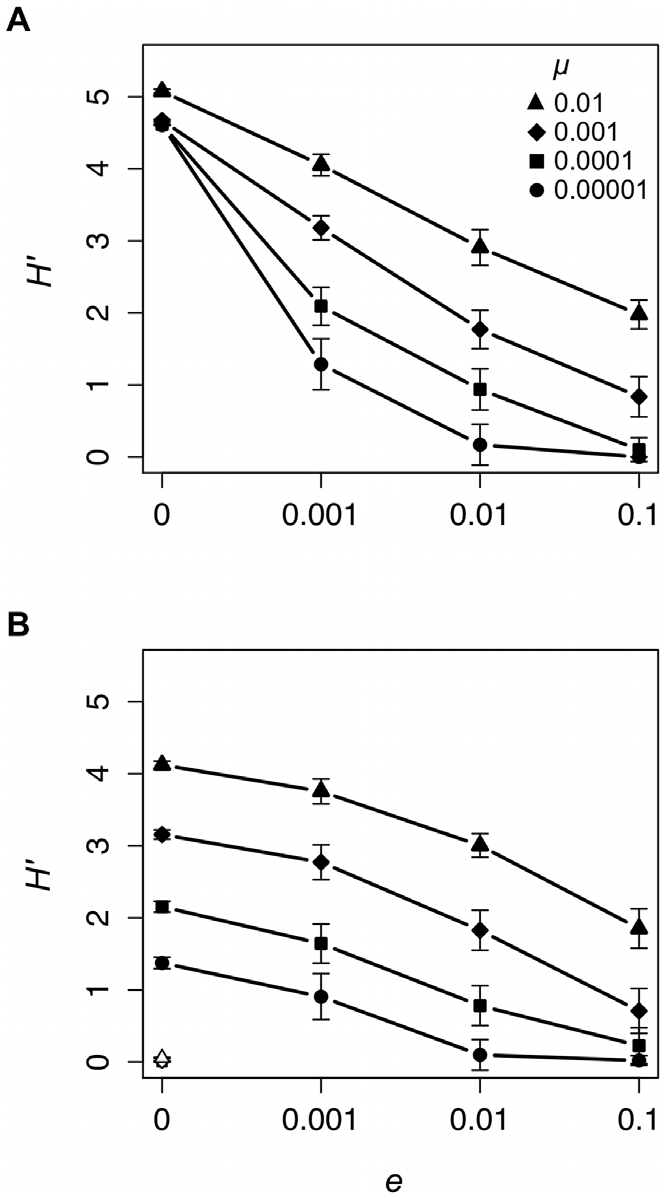
Local extinction rate (*e*) affects total cultural diversity as measured by Shannon's *H′* under equilibrium (A) and non-equilibrium (B) conditions. Each data point provides the mean ±1 standard deviation of 20 unique simulated populations. Black symbols represent data collected from populations with unbiased cultural transmission and white symbols data collected from populations with conformist cultural transmission.

Figure 3 summarizes the *H*′ results of our experiments. First, note that *H*′ increases with *μ*. Second, *e* has a significant effect on *H*′ under equilibrium (*μ* = 0.00001: *χ* ^2^ = 71.96, P<0.001; *μ* = 0.0001: *χ* ^2^ = 73.80, P<0.001; *μ* = 0.001: *χ* ^2^ = 73.56, P<0.001; *μ* = 0.01: *χ* ^2^ = 74.07, P<0.001) and non-equilibrium (*μ* = 0.00001: *χ* ^2^ = 66.53, P<0.001; *μ* = 0.0001: *χ* ^2^ = 70.76, P<0.001; *μ* = 0.001: *χ* ^2^ = 73.06, P<0.001; *μ* = 0.01: *χ* ^2^ = 73.64, P<0.001) conditions. Holding *μ* constant, frequent local extinction (*e* = 0.1) yields metapopulations that display substantially lower *H*′ than cases where there is no local extinction (*e* = 0). Conformist cultural transmission also yields low values of *H*′, even when there is no local extinction (Figure 3b).

**Figure 4:**
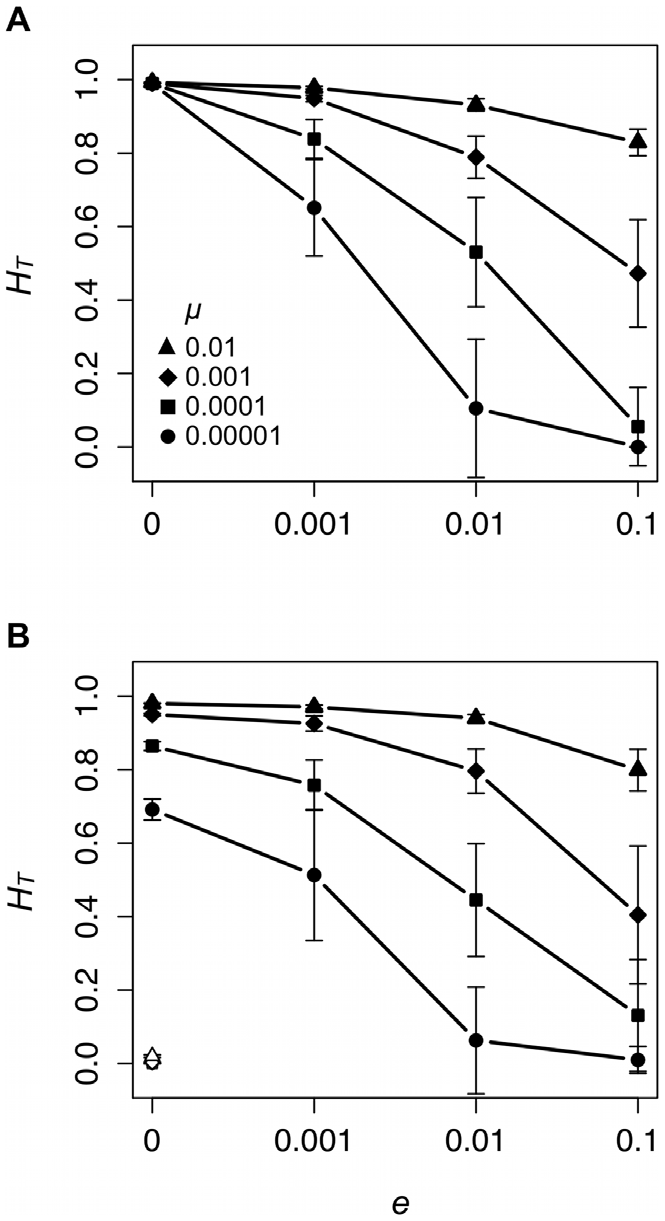
Local extinction rate (*e*) affects total cultural diversity as measured by *H_T_* under equilibrium (A) and non-equilibrium (B) conditions. Each data point provides the mean ±1 standard deviation of 20 unique simulated populations. Black symbols represent data collected from populations with unbiased cultural transmission and white symbols data collected from populations with conformist cultural transmission.

Although *H_T_* accounts for population structure while richness and *H*′ do not, all three measures provide similar pictures of how copying errors and local extinctions affect total cultural diversity. *H_T_* increases with *μ*, and *e* has a significant effect on *H_T_* under equilibrium (*μ* = 0.00001: *χ* ^2^ = 71.68, P<0.001; *μ* = 0.0001: *χ* ^2^ = 73.80, P<0.001; *μ* = 0.001: *χ* ^2^ = 73.35, P<0.001; *μ* = 0.01: *χ* ^2^ = 74.07, P<0.001) and non-equilibrium (*μ* = 0.00001: *χ* ^2^ = 64.62, P<0.001; *μ* = 0.0001: *χ* ^2^ = 69.84, P<0.001; *μ* = 0.001: *χ* ^2^ = 72.63, P<0.001; *μ* = 0.01: *χ* ^2^ = 73.93, P<0.001) conditions. *H_T_* decreases monotonically as *e* increases (Figure 4). And, as was the case for the other two measures of total diversity, frequent group extinctions (*e* = 0.1) and conformist transmission have similar consequences for *H_T_* (Figure 4b).

### Local extinction constrains group differentiation

Researchers are divided about the magnitude of geographic variation in Middle Paleolithic technological behavior. Some remark at the high level of regional diversity in stone artifacts (e.g., [8], [48]), whereas others emphasize the similarity of evidence across Eurasia (e.g., [49], [50]). Nonetheless, it is worth considering how the frequency of localized extinctions might act on geographic differentiation, or variation among spatially defined groups.

**Figure 5:**
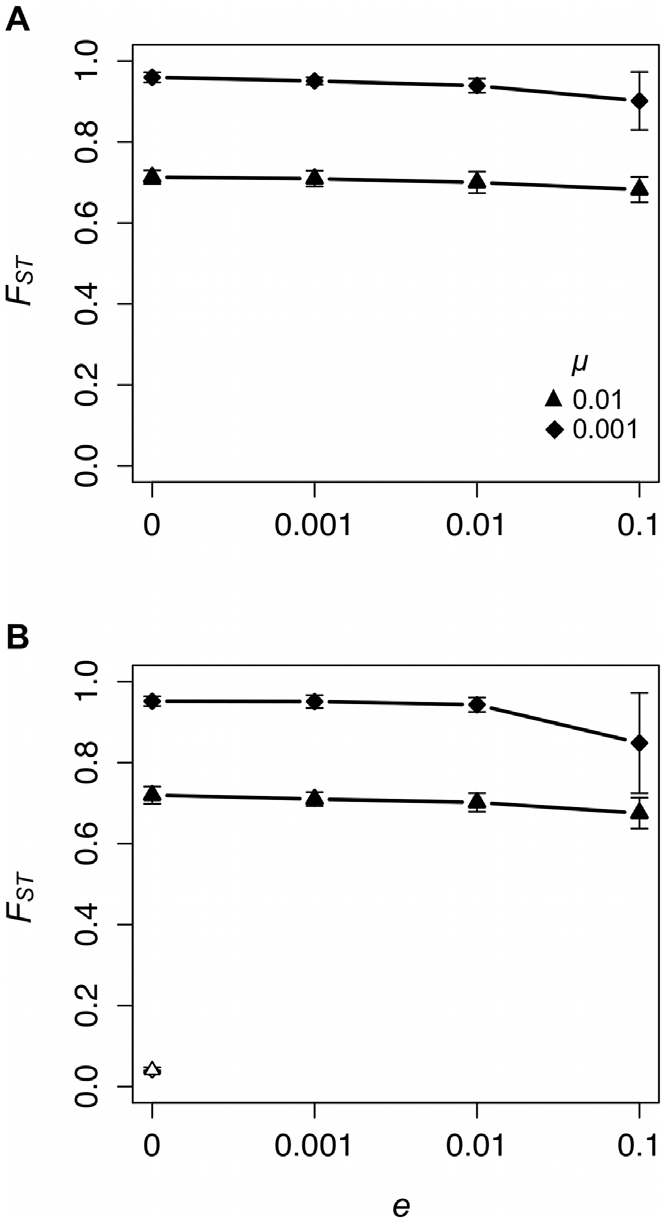
Local extinction rate (*e*) affects group differentiation as measured by *F_ST_* under equilibrium (A) and non-equilibrium (B) conditions. Each data point provides the mean ±1 standard deviation of 20 unique simulated populations. Black symbols represent data collected from populations with unbiased cultural transmission and white symbols data collected from populations with conformist cultural transmission.

The *F_ST_* results are summarized in Figure 5. Recall that *F_ST_* makes use of the relative frequencies of all cultural variants, not just the modal variant of each group. More importantly, *F_ST_* also takes into account the effect of *e* on *H_T_*. Two points are worthy of note. First, higher copying error rates yield lower *F_ST_* for all *e*. Second, *e* shows a significant effect on group differentiation under equilibrium (*μ* = 0.001: *χ* ^2^ = 25.22, P<0.001 and *μ* = 0.01: *χ* ^2^ = 12.77, P = 0.005) and non-equilibrium (*μ* = 0.001: *χ* ^2^ = 24.37, P<0.001 and *μ* = 0.01: *χ* ^2^ = 19.28, P<0.001) conditions. In our model, higher rates of local extinction constrain differentiation among groups as measured by *F_ST_*.

**Figure 6:**
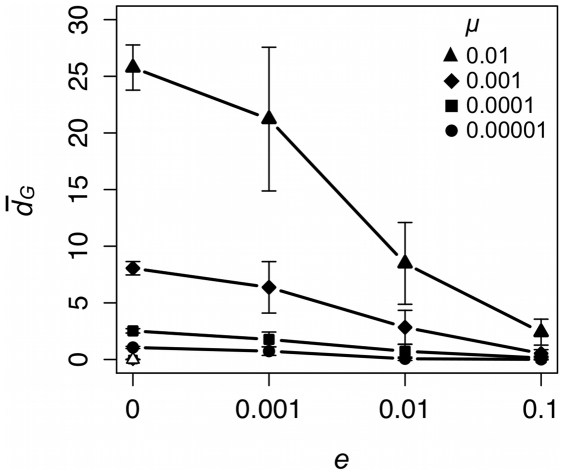
Local extinction rate (*e*) affects group differentiation as measured by 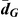. Each data point provides the mean ±1 standard deviation of 20 unique simulated populations. Black symbols represent data collected from populations with unbiased cultural transmission and white symbols data collected from populations with conformist cultural transmission.

Figure 6 summarizes the 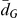 results for metapopulations simulated over different values of *μ* and *e*. In general, 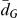 increases with *μ*. In addition, *e* has a significant effect on 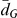 for all levels of *μ* tested (*μ* = 0.00001: *χ* ^2^ = 65.67, P<0.001; *μ* = 0.0001: *χ* ^2^ = 62.12, P<0.001; *μ* = 0.001: *χ* ^2^ = 66.19, P<0.001; *μ* = 0.01: *χ* ^2^ = 66.69, P<0.001). As was the case with *F_ST_*, the 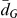 results show that there is less differentiation between groups in metapopulations plagued by a higher frequency of local extinctions (Figure 6). In short, the model predicts that group differentiation as measured by the mean distance between modal variants would decrease as the frequency of local extinction increases. It should be emphasized that this result assumes that cultural variants are neutral and have no effect on the probability of a local group going extinct.

Frequent local extinctions can decrease 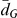 to levels similar to those that result from conformist cultural transmission with no local extinctions (Figure 6b). This is not the case with the other measure of group differentiation calculated here. With unbiased cultural transmission, increasing *e* does not bring the *F_ST_* values closer in line with those that result from conformist cultural transmission (Figure 5b). Given that metapopulations are initialized as perfectly homogeneous in the non-equilibrium version of our model, conformist transmission within groups yields a very low level of group differentiation even in the absence of local extinction. On this point, *F_ST_* and 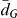 agree. One might reasonably predict that conformist transmission within groups should yield a *high* level of group differentiation regardless of how it is measured, but this prediction is likely to be met only when starting metapopulations are highly polymorphic and local extinctions are extremely rare (*e*≈0).

### Local extinction slows the rate of cumulative change in neutral cultural variants

**Figure 7:**
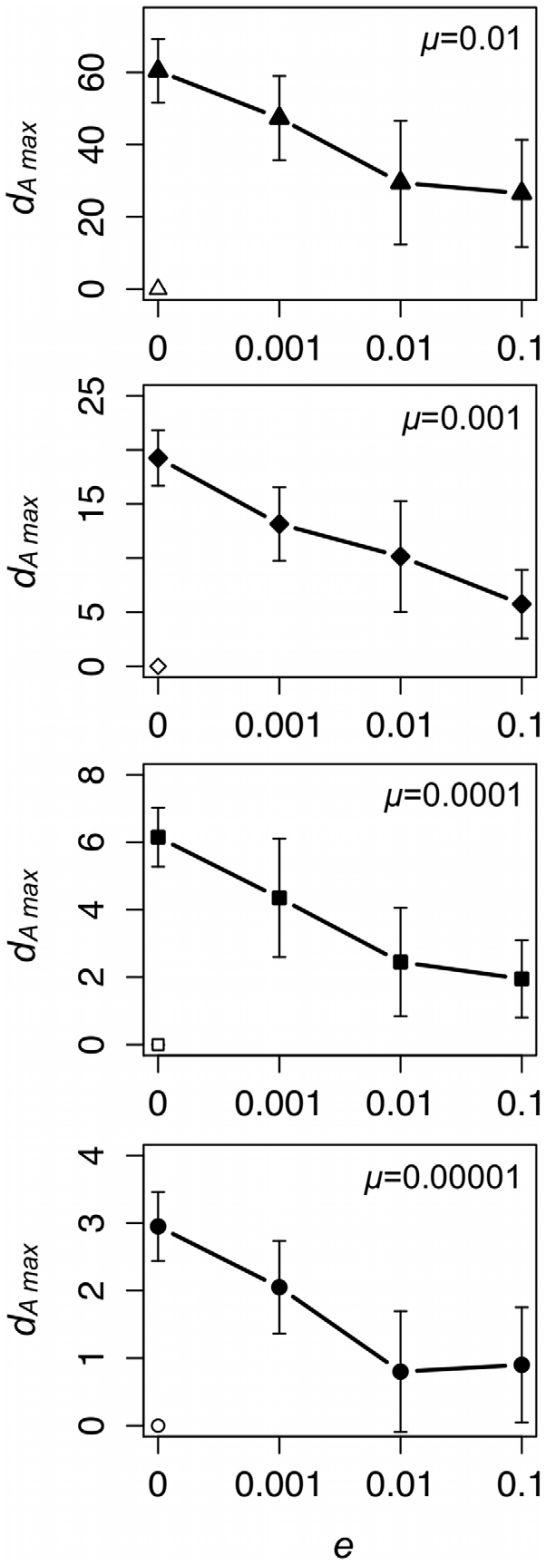
Local extinction rate (*e*) affects the rate of cumulative cultural change (*d_A max_*). Each data point provides the mean ±1 standard deviation of 20 unique simulated populations. Black symbols represent data collected from populations with unbiased cultural transmission and white symbols data collected from populations with conformist cultural transmission.

The results concerning the effect of *e* on *d_A max_*—and, by extension, the effect of local extinction and recolonization on the rate of neutral cumulative change per cultural transmission event—are summarized in Figure 7. As one would expect, there is a positive relationship between *d_A max_* and *μ*: the number of copying errors that accumulate in a metapopulation increases with the rate of copying error. More importantly, *e* has a significant effect on *d_A max_* for all *μ* tested (*μ* = 0.00001: *χ* ^2^ = 47.41, P<0.001; *μ* = 0.0001: *χ* ^2^ = 48.91, P<0.001; *μ* = 0.001: *χ* ^2^ = 52.52, P<0.001; *μ* = 0.01: *χ* ^2^ = 41.58, P<0.001), and this effect is negative. Given an equal number of cultural transmission events, metapopulations marked by frequent local extinctions retain *less* cumulative change per cultural transmission event than metapopulations that are demographically more robust (Figure 7). Finally, for the two lowest copying error rates, frequent local extinctions yield *d_A max_* values that are comparable, if not equivalent, to those one would expect to see with conformist transmission but no local extinction.

## Discussion

The model clearly shows that relatively frequent extinction of local groups could have important consequences for levels of diversification and rates of change in material culture. Holding *μ* constant, high rates of local group extinction would have the same effect on total cultural diversity, group differentiation (as is most commonly measured by archaeologists), and the rate of cumulative change as low copying error rates or—at least for some values of *μ*—conformist cultural transmission. In light of this, we may want to revisit the notions that Middle Paleolithic societies were marked by a drastically lower cognitive capacity for innovation and cultural change or by a long-lived and widespread conformist biased mechanism of cultural transmission. Perhaps the remarkable stability of Middle Paleolithic and earlier “cultures” is not to be found in their makers' capacities for innovation and change, but rather in the demographic fragility of the small social groups in which they lived.

To the best of our knowledge, this is the first attempt to model the effects of local extinction on the evolution of selectively neutral cultural traits. However, population geneticists have been studying the effect of local extinction on selectively neutral genetic diversity in structured populations for decades, and their findings provide important context for our results. For example, mathematical models show that increasing the rate of local extinction leads to a loss of neutral genetic diversity at the level of the metapopulation [51]–[53]. The effects of local extinctions on neutral genetic differences between subpopulations have also been studied formally. Wright [54] first proposed that a process of local extinction and recolonization should enhance the effects of genetic drift among local subpopulations, thereby increasing between-group differences. But subsequent work has shown that there are conditions in which local extinction and recolonization serves more like gene flow, redistributing genetic variation among groups and dampening the effects of drift among subpopulations [51], [55]–[58]. Under such conditions, increasing the frequency of local extinctions can actually reduce the level of genetic differentiation among local groups [55], [58].

Returning to cultural evolution, the results of the current study are largely complementary to work by Powell et al. [22], Shennan [21], [59], and Henrich [23], rather than confirmatory. Explaining why certain advantageous behaviors were widely and rapidly adopted in the late Middle Stone Age and early Upper Paleolithic does not explain why other behaviors, some apparently neutral, appear so stable and regionally homogeneous in earlier times. These other researchers are concerned mainly with the spread and persistence of complex behaviors that have positive fitness effects, whereas we have examined selectively neutral traits, characters with no consequences for the fitness of the individuals (or groups) displaying them. Furthermore, while their studies address the consequences of population size and connectivity (via migration) on cultural complexity, ours investigates the effect of local extinctions on cultural diversity and rates of change. Although the assumptions may differ between these models, their findings seem to underscore the same point: factors that influence a population's effective size are also likely to affect cultural complexity, diversity, and change.

We emphasize that the results of our model, or of any model for that matter, can definitively *disprove* only hypotheses that are built into them. This modeling exercise was designed to evaluate propositions concerning the effects of local extinctions on evidence for cultural diversity and change. Showing that this is possible provides a viable alternative or addition to existing explanations for long-standing observations about Lower and Middle Paleolithic cultures. In the end, the usefulness of this particular account rests on the strength of its assumptions, and its ability to account for the empirical record. As summarized above, there are several good reasons to suppose that local Lower and Middle Paleolithic groups frequently disappeared from the landscape. However, we emphasize that these findings cannot exclude arguments that Lower and Middle Paleolithic hominins differed cognitively from later human groups and that these differences explain the signals we see in the archaeological record. To the contrary, our results show that copying errors rates, which ultimately depend on cognitive capacities, have strong effects on diversity, group differentiation, and rates of change independent of local extinction rates. Likewise, the robustness of small local groups might well be tied to differences in biology, cognition, or behavior. Our results, like those of Powell et al. [22] and others, would shift the focus from cognitive characteristics related specifically to the production of material culture toward characteristics that would affect demographic stability.

Our findings concerning the effects of local extinctions on cultural diversity and regional differentiation may also be relevant to populations other than Lower and Middle Paleolithic societies. Archaeological and historical evidence indicate that more recent populations also experienced periods of profound demographic attrition that might well have involved stochastic extinction of local groups (e.g., [39], [60]). Populations expanding rapidly into previously un-colonized and unfamiliar landscapes, groups on small isolated islands, and people living in very harsh or rapidly fluctuating environments might all be at risk of losing many small local groups. This would have the same consequences for material culture as have been described here (see also [23]). For example, it is worth considering whether localized demographic instability of a colonizing population could have contributed to the apparent continental-scale homogeneity of archaeological “cultures” such as Clovis in North America, although the time frames involved are quite different from the cases considered here.

Further testing of our model would involve examining and comparing cases with different rates of local extinction (measured or inferred). However, there are added complications. In our model, copying error and local group extinction are not linked, implying that fidelity in transmission is independent of “stress” on subpopulations. In real world populations this may not be the case. We can imagine two opposing scenarios wherein fidelity of transmission and local extinction rates could be linked. On one hand, *μ* and *e* could be positively correlated, such that more stressful conditions and greater demographic instability increase the rate of copying errors. The “variability selection hypothesis” [61] provides one example of such a scenario. Boyd and Richerson have also described in depth how variable conditions could favor greater levels of experimentation in dual inheritance situations (e.g., [62]). Because copying error rates and local extinction rates act on cultural change and diversification in opposing ways, their effects might cancel out if they were positively correlated. On the other hand, *μ* and *e* could be negatively correlated, such that copying error rates tend to be lower in demographically unstable situations. If the fidelity of cultural transmission increased under demographic stress, the effects would be additive and we would expect much greater attenuation of change and restriction of diversity when times were hard.

In considering the implications of a simple and abstract model for a messy Pleistocene archaeological record, it is also important to be mindful of differences in scale. In this paper we examine diversity within a population of individuals. Archaeologists study diversity within and among assemblages of culture material created by many individuals over long spans of time. We do not have to worry about the conflation of time when calculating diversity within a population of contemporaneous individuals. When assessing diversity in archaeological assemblages, however, the effects of time averaging are important.

The model discussed here is most closely related to a formal model presented by Slatkin [55] (see his Model II with propagule pool mode of recolonization) with four important exceptions: we model cultural transmission rather than genetic transmission, variation is introduced via copying errors only, with no recombination, copying errors follow a symmetric random walk with a step length of 1 rather than an “infinite alleles” model of mutation, and our model is spatially explicit. Despite these differences, however, the two models provide similar predictions. We find that a pattern of local extinction and recolonization constrains total diversity and group differentiation and attenuates the rate of cumulative change per transmission event. Local extinction and recolonization may affect neutral cultural diversity differently when migration and/or intergroup cultural transmission are included in the model. For instance, there may be a threshold in the frequency of intergroup movement above which local extinction may increase total diversity, group differentiation, and rates of cumulative change relative to no local extinction. We can use simulation to estimate this intergroup transmission threshold for given values of *N* and *e*. Comparing simulated estimates of this intergroup transmission threshold to relative estimates of the “interconnectedness” of Lower and Middle Paleolithic societies drawn from archaeological data such as raw material movements (e.g., [63]) is the next logical step in testing the hypothesis that local extinction played a significant role in shaping the material record of the Lower and Middle Paleolithic.

## Supporting Information

Text S1 **Source code.** The downloadable file, premo_kuhn_2010.nlogo, runs in NetLogo versions 4.0 and 4.1. NetLogo is freely available from the World Wide Web.
(ZIP)

Text S2 **Model description.** The complete model description follows a standard protocol for describing agent-based models, and it includes directions for replicating our results.
(DOC)

## References

[1] Ambrose S (2001). Paleolithic technology and human evolution.. Science.

[2] Foley R, Lahr MM (2003). On stony ground: lithic technology, human evolution and the emergence of culture.. Evolutionary Anthropology.

[3] Hoffecker J (2005). Innovation and technological knowledge in the Upper Paleolithic of northern Eurasia.. Evolutionary Anthropology.

[4] Mithen S (1996). The Prehistory of the Mind..

[5] Boëda E (1991). Approche de la variabilite des systemes de production lithique des industries du Paleolithique inferieur et moyen: chronique d'une variabilite attendue.. Techniques et Culture.

[6] Delagnes A, Meignen L, Hovers E, Kuhn S (2005). Diversity of lithic production systems in the Middle Paleolithic in France. Are there any chronological trends.. Transitions before the Transition: Evolution and Stability in the Middle Paleolithic and Middle Stone Age.

[7] Meignen L, Delagnes A, Bourguignon L, Adams B, Blades B (2009). Patterns of lithic raw material procurement and transformation during the Middle Paleolithic in western Europe.. Lithic Materials and Paleolithic Societies.

[8] Mellars P (1996). The Neanderthal Legacy: An Archeological Perspective from Western Europe..

[9] Kuhn S, Stiner M, Panter-Brick C, Layton RH, Rowley-Conwy PA (2001). The antiquity of hunter-gatherers.. Hunter-Gatherers, an Interdisciplinary Perspective.

[10] Bocquet-Appel J-P, Tuffreau A (2009). Technological responses of Neanderthals to macroclimatic variations (240,000–40,000 BP).. Human Biology.

[11] Kandler A, Laland KN (2009). An investigation of the relationship between innovation and cultural diversity.. Theoretical Population Biology.

[12] O'Brien MJ, Shennan S, O'Brien M, Shennan S (2010). Issues in Anthropological studies of innovation.. Innovation in Cultural Systems: Contributions from Evolutionary Anthropology.

[13] Klein RG (2000). Archaeology and the evolution of human behavior.. Evolutionary Anthropology.

[14] Klein RG, Edgar B (2002). The Dawn of Human Culture..

[15] Wynn T, Coolidge FL (2004). The expert Neandertal mind.. Journal of Human Evolution.

[16] Gowlett J, Mellars P, Gibson K (1996). Mental abilities of early *Homo*: elements of constraint and choice in rule systems.. Modeling the Early Human Mind.

[17] Sharon G (2009). Acheulian giant-core technology: a worldwide perspective.. Current Anthropology.

[18] McNabb J, Binyon F, Hazelwood L (2004). The large cutting tools from the South African Acheulean and the question of social traditions.. Current Anthropology.

[19] Roebroeks W (2006). The human colonisation of Europe: where are we?. Journal of Quaternary Science.

[20] Dennell RW, Martinón-Torres M, Bermudez de Castro JM (2010). Hominin variability, climatic instability and population demography in Middle Pleistocene Europe.. Quaternary Science Reviews in press.

[21] Shennan S (2000). Population, culture history, and the dynamics of culture change.. Current Anthropology.

[22] Powell A, Shennan S, Thomas MG (2009). Late Pleistocene demography and the appearance of modern human behavior.. Science.

[23] Henrich J (2004). Demography and Cultural Evolution: Why adaptive cultural processes produced maladaptive losses in Tasmania.. American Antiquity.

[24] Lycett S, Norton C (2010). A demographic model for Palaeolithic technological evolution: The case of East Asia and the Movius Line.. Quaternary International.

[25] Richerson P, Boyd R, Bettinger R (2009). Cultural innovations and demographic change.. Human Biology.

[26] Henrich J, O'Brien M, Shennan S (2010). The evolution of innovation-enhancing institutions.. Innovation in Cultural Systems: Contributions from Evolutionary Anthropology.

[27] Eren MI, Greenspan A, Sampson CG (2008). Are Upper Paleolithic blade cores more productive than Middle Paleolithic discoidal cores? A replication experiment.. Journal of Human Evolution.

[28] Noonan J, Coop G, Kudaravalli S, Smith D, Krause J (2006). Sequencing and analysis of Neanderthal genomic DNA.. Science.

[29] Green R, Malaspinas A, Krause J, Briggs A, Johnson P (2008). Complete Neandertal mitochondrial genome sequence determined by high-throughput sequencing.. Cell.

[30] Briggs AW, Good JM, Green RE, Krause J, Maricic T (2009). Targeted retrieval and analysis of five Neandertal mtDNA genomes.. Science.

[31] Richards M, Pettitt P, Stiner MC, Trinkaus E (2001). Stable isotope evidence for increasing dietary breadth in the European mid-Upper Paleolithic.. Proceedings of the National Academy of Sciences.

[32] Stiner M (2002). Carnivory, coevolution, and the geographic spread of the genus *Homo*.. Journal of Archaeological Research.

[33] Kuhn S, Stiner M (2006). What's a mother to do? A hypothesis about the division of labor among Neandertals and modern humans in Eurasia.. Current Anthropology.

[34] Hocket B, Haws J (2004). Nutritional ecology and the human demography of Neanderthal extinction.. Quaternary International.

[35] Diamond JM, Nitkei MH (1984). “Normal” extinctions of isolated populations..

[36] Belovsky G, Soulé ME (1987). Extinction models and mammalian persistence.. Viable Populations of Conservation.

[37] McKinney ML (1997). Extinction vulnerability and selectivity: combining ecological and paleontological views.. Annual Review of Ecology and Systematics.

[38] Gittleman JL, Anderson CG, Cates SE, Luh H, Smith JD, McKinney M, Drake J (1997). Detecting ecological pattern in phylogenies.. Biodiversity Dynamics: Turnover of Populations, Taxa, and Communities.

[39] Riede F (2009). Climate and demography in early prehistory: using calibrated ^14^C dates as population proxies.. Human Biology.

[40] Hublin J-J, Roebroeks W (2009). Ebb and flow or regional extinctions?. C R Palevol.

[41] Wilensky U (1999). NetLogo.. http://ccl.northwestern.edu/netlogo/.

[42] Grimm V, Berger U, Bastiansen F, Eliassen S, Ginot V (2006). A standard protocol for describing individual-based and agent-based models.. Ecological Modelling.

[43] Nei M, Kumar S (2000). Molecular Evolution and Phylogenetics..

[44] Wright S (1943). Isolation by distance.. Genetics.

[45] Wright S (1951). The genetical structure of populations.. Annal of Eugenics.

[46] Wright S (1965). The interpretation of population structure by F-statistics with special regard to systems of mating.. Evolution.

[47] Nei M (1973). Analysis of gene diversity in subdivided populations.. Proceedings of the National Academy of Sciences.

[48] Foley R, Gamble C (2009). The ecology of social transitions in human evolution.. Philosophical Transactions of the Royal Society B.

[49] Gamble C (1999). The Paleolithic Societies of Europe..

[50] Klein RG (1999). The Human Career..

[51] Wade MJ, McCauley DE (1988). Extinction and recolonization: Their effects on the genetic differentiation of local populations.. Evolution.

[52] McCauley DE (1991). Genetic consequences of local population extinction and recolonization.. Trends in Ecology and Evolution.

[53] Whitlock MC, Barton NH (1997). The effective size of a subdivided population.. Genetics.

[54] Wright S (1940). Breeding structure of populations in relation to speciation.. American Naturalist.

[55] Slatkin M (1977). Gene flow and genetic drift in a species subject to frequent local extinctions.. Theoretical Population Biology.

[56] Slatkin M (1985). Gene flow in natural populations.. Annual Review of Ecology and Systematics.

[57] Slatkin M (1987). Gene flow and the geographic structure of natural populations.. Science.

[58] Whitlock MC, McCauley DE (1990). Some population genetic consequences of colony reformation and extinction: Genetic correlations within founding groups.. Evolution.

[59] Shennan S (2001). Demography and cultural innovation: a model and its implications for the emergence of modern human culture.. Cambridge Archaeological Journal.

[60] Chamberlain A (2009). Archaeological demography.. Human Biology.

[61] Potts R (1998). Variability selection in hominid evolution.. Evolutionary Anthropology.

[62] Boyd R, Richerson P (2005). The Origin and Evolution of Cultures..

[63] Whallon R (2006). Social networks and information: Non-“utilitarian” mobility among hunter-gatherers.. Journal of Anthropological Archaeology.

