# Supplementary material for: Modeling Effects of Local Extinctions on Culture Change and Diversity in the Paleolithic
Source: PLoS One. 2010 Dec 17;5(12):e15582. doi: 10.1371/journal.pone.0015582 (PMC3003693; doi:10.1371/journal.pone.0015582)
Supplement: Text S2 — Model description. The complete model description follows a standard protocol for describing agent-based models, and it includes directions for replicating our results. (DOC) [file pone.0015582.s002.doc]

**Text S2. Model Description**

**Purpose**

This purpose of this model is to study the effect of local (or group) extinction on the evolution of neutral cultural diversity in artificial societies of individuals who pass cultural variants via social learning. We simulate metapopulations over different group extinction rates to study the effect of local extinctions on total diversity, long-term cumulative culture change, and regional cultural differentiation in spatially structured populations of constant size and density. The results show an inverse relationship between the frequency of local extinctions and the amount and rate of culture change through time. Frequent local extinctions also constrain differentiation between groups.

**Agent classes, variables, & temporal and spatial scales**

Our model employs two classes of agents: individuals and groups. Individuals serve as the vehicles of cultural transmission. Individuals have only 3 variables: one representing age (*age*), one representing the probability of making a copying error when learning (*errorProb*), and one representing a neutral cultural variant (*culturalVariant*). *age* can be assigned one of two possible values: 0 (born during the current time step) or 1 (born during the previous time step). *errorProb* is given by a real number bound by 0 and 1. *culturalVariant* can take the value of any integer. Individuals have only two methods: *die* and *learn*. An individual dies if its group goes extinct or its *age*=1 at the end of a time step. The method, *learn*, defines the way in which each naive “offspring” (i.e., and individual with *age*=0) acquires its *culturalVariant* from a member of the “parental” generation (i.e., an individual with *age*=1). This model implements 2 mechanisms of cultural transmission: unbiased and conformist biased transmission (see Submodels).

In the context of this model, groups are best described as spatially explicit containers of individuals. Groups provide the population with spatial structure. Each group is characterized by a unique Cartesian coordinate, a group extinction probability (*groupExtinctionProb*), which is represented by a real number bound by 0 and 1, and *modeCulturalVariant*, which contains the mode of the variants displayed by its members. Groups have only one method, called *fission*.

The model proceeds in simulated time steps (or *ticks*). Each time step represents a single generation: by the end of each time step, the entire “parental” generation--those individuals alive at the beginning of the time step--has been replaced by a new generation of “offspring.”

The model is spatially explicit. The spatial environment is a grid of regularly spaced and shaped square cells. The opposite edges of the grid are wrapped to avoid edge effects. Although cell size does not equate to an empirical value, one can think of it as the area needed to support the subsistence activities of a single social group (*N*=25) within an idealized homogeneous landscape. Thus, each cell contains a single group, which can contain up to *N* individuals.

**Process Overview and Scheduling**

Each time step is composed of 5 distinct processes, which occur in the following order: individuals get older, groups are exposed to risk of extinction, the “offspring” generation is created, cultural variants are transmitted from the “parental” generation to the “offspring” generation, and the “parental” generation is culled.

At the very beginning of each time step, all living individuals get older. They update the value of their *age* variable from 0 to 1 to reflect the fact that they are now part of the “parental” generation and can serve as teachers later in the time step.

Second, all groups are exposed to localized extinction. If a real number chosen randomly from a uniform distribution bound by 0 and 1 is less than or equal to a group’s *groupExtinctionProb*, then the individuals who are members of that group are “killed,” and they are removed from the simulation immediately. Following a local extinction event, the cell is empty. Each empty cell is repopulated with members of the “parental” generation taken from a randomly chosen neighboring group [see fission in Submodels]). Empty cells are repopulated after all groups have been exposed to a chance of local extinction.

Third, the “offspring” generation is created, which means that 25 new individuals are created in each group. Members of the “offspring” generation are distinguished from those that remain from the “parental” generation by their *age* (0 for offspring generation, 1 for parental generation) and by the fact that they have not yet adopted a cultural variant.

Fourth, all members of the newly created “offspring” generation undergo cultural transmission. The model implements two very different mechanisms of cultural transmission: unbiased and conformist biased cultural transmission. In both cases, however, cultural transmission occurs *within* groups only. There is no form of intergroup cultural transmission in the model aside from the movement of “colonizers” into newly empty cells. With unbiased cultural transmission, each “offspring” learns (i.e., copies) the variant expressed by its “teacher.” Each “offspring” randomly selects its teacher (with replacement) from among the “parental” generation survivors who inhabit the same group that the “offspring” occupies. With conformist biased transmission, the “offspring” does not learn from a “teacher,” but rather it adopts the cultural variant that is most common (i.e., the modal variant) among the “parental” generation survivors who inhabit the same group as the “offspring.” In the event that there is more than one mode in the variants displayed by the parental generation in a group, each naïve individual copies one of the modal variants chosen at random. Cultural transmission is noisy. The copying error rate, *errorProb*, provides the probability that an “offspring” makes a mistake when acquiring its variant during cultural transmission. Regardless of the mechanism of cultural transmission, copying errors result in the “offspring” adopting a variant that is either one integer larger or one integer smaller than the integer it was trying to copy. In this sense, copying error follows a one-dimensional random walk with a step length of 1. See submodels for more details on cultural transmission and copying errors.

Finally, all remaining members of the “parental” generation (i.e., *age*=1) are culled from the simulation. As a result, the population size (in this case, 2500 individuals) at the end of a time step is the same as it was at the beginning of the time step, but all of the individuals belong to the “offspring” generation (*age*=0).

**Design Concepts**

Evolution. In the context of this model, evolution refers to change in cultural variant frequencies in a structured population through time. Because cultural variants are selectively neutral (i.e., non-adaptive) in this model, the results speak only to neutral evolution.

Stochasticity. Stochasticity is incorporated into many aspects of this nondeterministic model: copying errors and local extinctions are represented probabilistically, agent order is randomized before every process, teachers are chosen randomly from among the set of eligible candidates, and the groups tabbed to fission in order to recolonize an empty cell are chosen randomly from among the empty cell’s 8 Moore neighbors.

**Initialization**

At the start of each simulation, all groups (*n*=100) possess the maximum number of individuals (*N*=25), for a total of 100 * 25 = 2500 individuals. It is preferable to assess demographic effects on diversity in systems that are at equilibrium. In the absence of selection, cultural diversity reaches equilibrium when the rate that new variants are introduced via copying errors matches the rate they are lost to drift. The number of generations required to reach equilibrium varies with population size, copying error rate, population structure, and, in our model, the frequency of local extinctions. When the copying error rate is very low, there are no local extinctions, and the starting population is perfectly homogeneous (i.e., all individuals display *culturalVariant*=0 at *t*_0_), many hundreds of thousands of generations must pass before cultural diversity approaches equilibrium. Metapopulations reach equilibrium more quickly if every individual displays a unique value at *culturalVariant* (in this case, an integer chosen randomly from between 0 and 10^8^) at the start of the simulation. When starting with heterogeneous metapopulations, we found that 50,000 time steps were sufficient for cultural diversity to reach equilibrium for all combinations of *µ* and *e* tested here.

In addition to data collected under equilibrium conditions, we analyze data collected from metapopulations that are not at equilibrium. There are two reasons for this. Starting from heterogeneous metapopulations complicates the task of measuring the effects of local extinctions on distance-based measures of group differentiation and cultural change, especially when *groupExtinctionProb* is very low or zero. A less pragmatic but no less important issue is that, while equilibrium serves as a useful convention for providing a clearer understanding of the effect of local extinctions on some diversity measures, it is not meant to reflect reality. It is not known whether cultural diversity ever reached equilibrium in Lower and Middle Paleolithic societies, nor is this assumption necessary here. The non-equilibrium data were collected after 100,000 time steps from metapopulations in which every individual displayed a *culturalVariant* of “0” at the start of the simulation. This complementary set of experiments allows us to study the effects of local extinctions on diversity measures, distance-based measures of group differentiation, and rates of cultural change as a function of time.

The table below provides the values used to initialize the simulations for the equilibrium and non-equilibrium experiments.

Parameter values used to initialize the simulations.

| Standard parameters |  |
| --- | --- |
| maxGroupSize (*N*) | 25 |
|  |  |
| Experimental parameters |  |
| groupExtinctionProb (*e*) | 0, 0.001, 0.01, 0.1 |
| errorProb (*µ*) | 0.00001, 0.0001, 0.001, 0.01 |
| conformistLearning | On, Off |
|  |  |
| Random number seeds |  |
| seed | 1, 2, 3, 4, 5, 6, 7, 8, 9, 10, 11, |
|  | 12, 13, 14, 15, 16, 17, 18, 19, 20 |

**Data Collection**

Data are collected at the end of each simulation. We collect data at the level of the group and the level of the metapopulation. From each group, we collect the modal cultural variant (*d_A_*), the mean difference between its modal variant and all other groups’ modal variants ($d_{G}$), and Shannon’s *H´*. At the level of the metapopulation, we collect variant richness (the total number of unique cultural variants displayed by the entire population), Shannon’s *H´*, total diversity (*H_T_*), within-group diversity (*H_S_*), maximum distance from ancestral (*d_A max_*), and the mean of the groups’ difference from all other groups ($\bar{d}_{G}$).

**Submodels**

Group dynamics: local extinction and *fission*. As described above, when a group undergoes a localized extinction event, all of the individuals within it are removed from the simulation. To repopulate the empty cell with some members of the “parental” generation who will serve as possible teachers for the next generation in that location, a group from the empty cell’s Moore neighborhood is randomly chosen to fission. Aside from being adjacent to the empty group, the only other requirement for serving as a source of “colonizers” is that the group must have at least 2 members. Once chosen, the group sends half of its individuals (rounded to the nearest whole number) to the empty cell.

Cultural transmission (*learn*). All cultural variants are selectively neutral. All cultural transmission occurs *within* spatially defined groups between the “parental” generation and the “offspring” generation. The model implements two mechanisms of cultural transmission (unbiased transmission and conformist biased transmission) although each simulation run can involve only one or the other.

Every unbiased cultural transmission event involves a naïve “offspring” who has yet to adopt a *culturalVariant* and an experienced teacher from the “parental” generation. The “offspring” copies the *culturalVariant* expressed by its teacher. Each “offspring” randomly chooses its teacher (with replacement) from the set of individuals who are members of the “parental” generation and members of the spatially defined group of which the “offspring” is currently a member.

Conformist cultural transmission works differently. With conformist biased transmission, the naïve “offspring” copies the variant that is most commonly displayed by the members of the “parental” generation who reside in its group. In cases where there are two or more modal variants, the “offspring” chooses one of them randomly.

Regardless of which mechanism of cultural transmission is used, naïve individuals do not copy variants perfectly. A naïve individual makes a mistake when copying a variant with probability *errorProb*. Copying errors always result in the “offspring” adopting a cultural variant that is either one step greater than or less than the integer it was trying to copy. In the parlance of population genetics, copying errors follow a single stepwise mutation model.

**Noteworthy model assumptions**

Our model assumptions are purposefully conservative. Here, we briefly discuss some of the more important assumptions of the present version of the model.

The model does not allow a naive individual to learn from a member of a different spatially defined group--cultural transmission occurs *within* groups only. Allowing for cultural variants to be transmitted across group boundaries, for example, would likely affect the results reported here. We would expect the magnitude of the effect of local group extinction on cultural diversity to decrease as the rate of intergroup transmission increased. Still, we would expect that our general finding--that frequent local group extinctions decrease the amount and rate of neutral cultural change and inhibit regional differentiation between groups--would hold true for low levels of intergroup transmission. However, it may be that, in the presence of non-zero intergroup transmission rates, *groupExtinctionProb* would need to be higher than what we tested here in order to show similar effects on cultural diversity.

This model also does not allow for horizontal cultural transmission: individuals can only learn from members of the previous generation, never from members of their own generation. It is unclear how including horizontal transmission would affect our results, and this might be worth investigating further.

Copying errors follow what population geneticists call a single stepwise mutation model. This kind of mutation model is often used to study neutral microsatellite, or STR (Short Tandem Repeat), loci in population genetics. Modifying the way in which copying errors affect cultural variants--for example, by using an “infinite alleles” approach instead of the single stepwise model or by only allowing copying errors to increase cultural variants in all cases--may yield results that differ from those reported in the text. However, it should be noted that using an infinite alleles approach may complicate the task of measuring change and that using a unidirectional stepwise approach may not allow one to make use of *F_ST_*.

**Source code**

Of course, the best description of any computer model is the program itself. Please see additional online supporting material or contact the corresponding author to obtain the source code for this model, which requires NetLogo (versions 4.0 or 4.1). NetLogo is freely available for download from the web (http://ccl.northwestern.edu/netlogo/). Once NetLogo is installed and the source code file is downloaded, simply double click on premo_kuhn_2010.nlogo to open the model. One can access the source code by clicking on the “Procedures” tab at the top of the NetLogo user interface. A copy of this model description also appears under the “Information” tab, along with directions on how to operate the simulation and replicate the results reported in the main text.
